# Exploring the health and well-being benefits of reduced working hours with maintained salary: A scoping review and evidence map

**DOI:** 10.5271/sjweh.4266

**Published:** 2026-03-01

**Authors:** Mireia Utzet, Mercè Soler, Jose Maria Ramada, Marta Menéndez, Michael Silva-Peñaherrera, Fernando G Benavides, Consol Serra

**Affiliations:** 1 Centre for Research in Occupational Health, Department of Medicine and Life Sciences, Universitat Pompeu Fabra, Barcelona, Spain.; 2 HMRI-Hospital del Mar Research Institute, Barcelona, Spain.; 3 CIBER of Epidemiology and Public Health, Madrid, Spain.; 4 Health Technology Assessment in Primary Care and Mental Health (PRISMA), Institut de Recerca Sant Joan de Déu, Esplugues del Llobregat, Barcelona, Spain.; 5 Occupational Health Service, Parc de Salut Mar, Barcelona, Spain.; 6 La Isla Network, Washington, USA.

**Keywords:** gender perspective, general health, mental health, occupational health, working time reduction, work–life balance

## Abstract

**Objectives:**

The aim of this review was to map the characteristics and the effects of interventions that reduce working hours with full pay maintained on workers’ health, well-being and work–life balance and to assess whether a gender perspective was incorporated.

**Methods:**

A scoping review was conducted following PRISMA-ScR and JBI guidelines. Scientific databases (PubMed, Scopus, Web of Science, PsycINFO, CINAHL, Cochrane, ProQuest, Epistemonikos) and grey literature sources (international, European and national labor and occupational health agencies) were systematically searched for studies published between April 2014 and May 2025 in English or Spanish. Eligible studies comprised employed adults in Scandinavian and Western European countries, the United States, Canada, Australia and New Zealand exposed to interventions reducing working hours with full pay maintained. Screening, data extraction, and quality appraisal were conducted independently. An evidence map was developed to synthesize the findings.

**Results:**

Ten scientific articles and five grey literature reports were included, seven from Scandinavian, seven from Western European countries, and one that included both regions. Working time reductions ranged from 10–25%. Most studies reported positive effects on work–life balance (100%), mental health (81.8%), and general health and well-being (58.3%). Qualitative data confirmed improvements in recovery, fatigue, and family time. Eleven studies included a gender perspective, with eight providing stratified analyses. Several studies indicated that women increased the time dedicated to caregiving and household, reinforcing traditional roles, while men’s involvement rose slightly without shifting responsibility equity.

**Conclusions:**

Despite heterogeneity of interventions and limited implementation contexts, it seems that reducing working hours to around 30–35 per week without pay loss may improve work–life balance, health, and well-being. Gender differences emerged, with women often facing increased unpaid work. As the evidence is still scarce particularly regarding long-term effects, sector-specific interventions, and gendered effects, further research is needed to inform and evaluate policies that promote equitable and sustainable work-time arrangements.

Paid work is a significant social determinant of health, offering potential benefits for social and psychological well-being but also posing risks, depending on the organization of work, employment conditions, and social context (1). A central element in work organization is the working time arrangement, both in terms of the number of working hours and the structure of the workday, which is crucial for ensuring decent work (2, 3). In fact, since 1919, the International Labor Organisation (ILO) has emphasized the importance of appropriate working hours for decent employment (4). In the European context, the maximum number of hours per working week is 48 hours (including ordinary working hours and overtime) (5). Nevertheless, the shift towards increased labor-market flexibility and a “24-hour” economy has led to extended work hours, increased overtime, more individualized and irregular work schedules (6); exacerbating the “permanent availability” and work–life conflicts, especially for low-skilled women who often face unpredictable or “asocial” hours (7).

The relationship between working time (both duration and organization) and health has been widely studied, showing long working hours (>55 hours per week) as a significant occupational health hazard, leading to increased risks of cardiovascular diseases like stroke and ischemic heart disease, as well as mental health issues (8, 9). Over the past century, the reduction of working hours has periodically re-emerged in European public debate—from early 20^th^-century labor movements advocating for the eight-hour day to work-sharing policies aimed at reducing unemployment during the 1980s and 1990s. For about a decade now, the reduction of working hours has been back in the political debate (10). In various European countries, pilot tests have been carried out to reduce working hours, promoted by national agreements [in Nordic countries (11)], by collective bargaining [in two Italian companies in the automotive sector (12)], or by the Autonomy think tank [including Iceland, Portugal and the The UK (13)]. However, research on the health impacts of reduced working hours remains limited, shows heterogeneous results and might be influenced by selective reporting of favorable outcomes. In 2019, a systematic review showed some evidence that reducing work hours with salary maintenance might decrease stress and improve sleep quality. However, this review was restricted to the Scandinavian context (with its own typology of social welfare state, characterized by fairly regulated labor markets or dual earner/dual carer family models), excluded grey literature, and lacked a gender-based analysis (14).

Furthermore, working time is a fundamental element for work–life balance, since, together with wages, it is one of the working conditions that has the greatest impact on the daily lives of the working population and their families. Balancing working time with personal, familial, and social needs is essential (15, 16). Working-time arrangements must balance organizational productivity needs with workers’ care, social, and rest requirements. Consequently, working time often highlights a fundamental gender gap. Concepts such as “double presence” or “worklife conflict,” which refer to the need to meet both salaried job demands and domestic responsibilities simultaneously, mainly affect women (17). Part-time work has frequently been presented as a “voluntary” solution to balance paid work and care time, often performed by women, but it carries precarious implications (18). Reducing working hours while maintaining salaries could help reconcile personal life (including care and leisure time) and paid work, potentially addressing gender inequalities within the structural conflict between business productivity and personal time. However, some studies suggest that men do not necessarily allocate their additional time off to caregiving or domestic responsibilities (19).

This study aims to map the characteristics and effects of interventions that reduce working hours while maintaining wages on workers’ general and mental health, well-being, and work–life balance, including both scientific and grey literature, to obtain a complete and detailed picture. Likewise, examine the extent to which gender perspective is integrated into the scientific and grey literature is essential to better understand how these measures may affect men and women differently, and thus contribute to a more equitable and realistic analysis of the observed results.

The aims of this review are to: (i) identify and map the interventions implemented to reduce working hours while maintaining salary, as well as the effects on workers’ general and mental health, wellbeing and work–life balance; (ii) analyze the extent to which the scientific and grey literature incorporate the gender perspective; and (iii) identify research gaps in the existing literature, highlighting areas for future research and practice.

## Methods

The Joanna Briggs Institute (JBI) and PRISMA-ScR guidelines were followed (20, 21). The protocol was registered on the Open Science Framework in October 2024 (https://osf.io/ymzvh). The study was designed including the gender perspective.

*Eligibility criteria.* The inclusion and exclusion criteria were defined according to the population, concept, and context (PCC) framework. The population comprised employed adults aged 16–65 years. Informal and unpaid workers were excluded for reasons of accessibility. The concept was defined as interventions to reduce working hours while maintaining wages and their effects on general health and wellbeing, categorized as general health, physical health and wellbeing, mental health, and work–life balance. Excluded interventions included those focused on partial or gradual return to work or adapted for health, pregnancy or personal family reasons, part-time or condensed working days (ie, working days that include the same number of hours per week in fewer days). The context was the workplace, including all sectors in countries considered Western (Scandinavian and Western European countries, the United States, Canada, Australia and New Zealand). Both scientific and grey literature (22) published between April 2014 and May 2025 in English or Spanish were included. Scientific literature excluded opinion articles, editorials, reviews, and conference abstracts. For the grey literature, annuals, books, chapters, reports, and technical documents were included, and presentations, press releases, datasets, tables, podcasts, and summaries were excluded.

*Research question.* The review question was: “What evidence exists regarding the effects on general health, mental health, wellbeing and work–life balance of interventions that reduce working hours while maintaining wages among employed adults?”

*Search.* The search of the scientific literature was carried out in PubMed, Web of Science, Scopus, Cochrane Library, PsycINFO, CINAHL, Epistemonikos and ProQuest. The SearchRefinery tool from the SR-Accelerator page was used to establish the search strategy. Specific descriptors and qualifiers from the thesaurus of the PubMed database were used for greater precision. Filters were applied by year of publication (studies published between April 2014 and April 2024) and language (English or Spanish). The search strategy was formulated for PubMed and adapted for use in other databases (see supplementary material, www.sjweh.fi/article/4266, table S1). The refined search was carried out on 24 May 2024. For the search of grey literature, a list of official organizations related to the labor field was elaborated. This list was made at three levels (see supplementary table S2): (i) global labor agencies and organizations, (ii) European-level labor agencies and organizations [for example, the European Agency for Safety and Health at Work (EU-OSHA)], and (iii) governmental agencies and organizations at the Western country level, specifically Scandinavian and Western Europe, Australia, New Zealand, Canada and the United States. A manual search was conducted by visiting the websites of the selected organizations and individually downloading the relevant grey-literature documents. We considered them relevant if they included one or more of the key terms from the literature search strategy (eg, “reduced working hours”), were published in the last ten years (April 2014–April 2024), and in the selected languages (English or Spanish). This search was carried out 20–24 May 2024.

An updated search was conducted on 15 May 2025, to include both, scientific and grey literature, published between April 2014 and 15 May 2025.

*Selection.* Evidence selection was performed independently in pairs by four investigators using the Rayyan systematic review software (23). Initially, four investigators individually reviewed the titles and abstracts of 5% to confirm the inclusion and exclusion criteria and resolve any doubts that might arise at this stage. Subsequently, the screening of titles and abstracts by pairs was carried out. The articles that met the inclusion criteria passed to the full-text screening phase, also by pairs. In this phase, the full text was reviewed to decide the final inclusion or exclusion. In case of discrepancies, they were discussed to reach an agreement. From this point onward, both the scientific and grey literature will be collectively referred to as ‘included studies’ throughout the manuscript.

*Quality appraisal.* Quality appraisal was conducted using the mixed methods appraisal tool (MMAT), version 2018 (24), which was designed for systematic reviews encompassing different study designs. Each study was then assigned to the appropriate MMAT category based on its methodology, and five criteria specific to that category were rated. As recommended by authors, detailed ratings are presented for each criterion to thoroughly inform study quality (supplementary table S3). A quality score was retrieved, ranging from zero (meeting none of five criteria) to five (meeting all of criteria). Two authors independently assessed the quality of included reviews. Quality assessment was undertaken not to exclude studies but to inform the interpretation of results.

*Data extraction.* Five independent researchers extracted relevant data from each included study and summarized them using a pre-piloted Excel data extraction form that included (i) the main characteristics of the document (title, author(s), year of publication, type of document, organization publishing the document, and country); (ii) the program/intervention variables (study design, intervention description, percentage of time reduction, temporary or permanent reduction, voluntary or mandatory for the workers, the distribution of the reduced working hours (eg, one day per week, one hour per day), public sector involvement (yes/no), benefits or support provided during the intervention; sector and occupation) (iii) the study population, number of participants, age distribution, sex/gender distribution); (iv) socioeconomic characteristics of the country and political framework; (v) outcomes, that included variables (general health, physical health, mental health, work–life balance, and sick leave), results, and type of analyses (eg, adjusted, stratified); (vi) health and work beliefs; (vii) inclusion of gender perspective (in the study design and stratified results); (viii) limitations and (ix) recommendations. For qualitative results, the extracted outcomes were thematically categorized (general health, physical health, mental health, work–life balance, and sick leave). Within each category, qualitative findings were recorded, along with the type of analysis reported (eg, interviews conducted before and after the study). Afterwards, a crossed thorough review of the information collected was carried out to ensure its accuracy and completeness.

*Synthesis of results and evidence map.* Both qualitative and quantitative findings were collated, summarized and thematically reported in three tables, and an evidence map was developed. The evidence map was designed to enable users to interactively access information across multiple interventions and outcomes. The Tableau data visualization tool was used to render the map (25). In the synthesis, quantitative findings were reported in all cases, explicitly indicating whether they were statistically significant. In addition, all authors of the studies and reports included in this review were contacted to validate the results and ensure the accuracy and relevance of the findings.

*Gender perspective.* A gender perspective was incorporated throughout the study. Specifically, for data extraction, we assessed whether the studies stratified the analysis by gender or whether there were specific studies according to the work environment, such as, for example, industries or sectors dominated by women or men. Also, for the synthesis of results and their interpretation, we assessed whether the studies included gender perspective.

## Results

### Study selection

**Figure 1:**
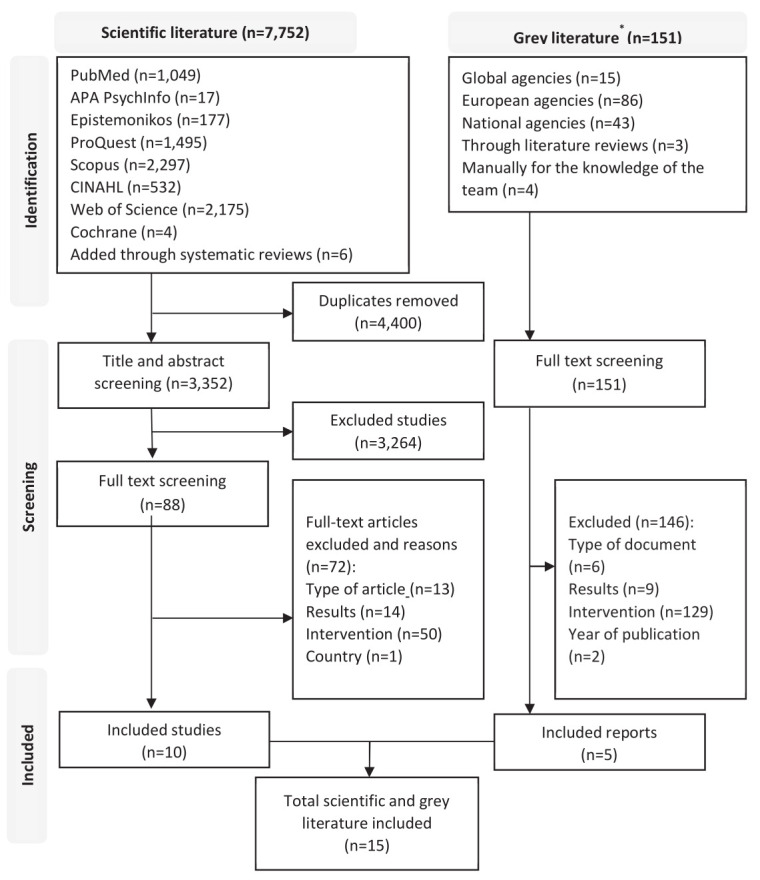
Flowchart of the identification, screening and selection of scientific and grey literature. * A list of government and labor agencies included for the grey literature search is reported in supplementary table S2.

Database searches identified 7752 citations. After removing duplicates, the title and abstract of 3352 articles were screened. A full-text review was conducted on 88 articles, and 10 were finally included. Grey literature searching identified 151 records for full text screening, and 5 reports were included (figure 1).

### Study characteristics

The summary of the included studies is presented in table 1. Publication years ranged from 2017 to 2025, with 67% (N=10) published from 2021 onwards. Study design included quasi-experimental studies (N=10) (26–35), cluster randomized controlled trials (N=2) (36, 37), one pre-post evaluation (38), and one cross-sectional study (N=1) (39). Three studies incorporated a qualitative approach (40, 31, 38). Geographic focus was predominantly on Scandinavian or Western European countries, with the majority conducted in Sweden (N=7, including five analyses on the same cohort) and the remaining studies based in France, Portugal, the UK, Iceland, Belgium and Spain. Sample size ranged from 12 (27) to 11 607 (28).

**Table 1:** Summary of scientific and grey literature studies included in the review. [ASÍ=Confederation of Icelandic Labour Unions (Alþýðusamband Íslands); DHEAS=dehydroepiandrosterone sulfate; CG=control group; IG=intervention group; NR=not reported; NS=not significant; QE=quasi experimental; RCT=randomised controlled trial; Strat=statification; SME=small and medium-sized enterprises].

Reference	Study design	Intervention & time period	Population and sample size	Analysis	Gender strat	Outcomes ^1^
Barck-Holst ^2^ 2017 Sweden (26)	QE	25% reduction (from 39 to 29 hours per week approx.) 18 months 2005-2006	Full-time workers of 7 public social service agencies. N=89: IG=12; CG=77	Multiple regression models to estimate differences in change over time in IG vs. CG. Models adjusted for baseline values, gender and age.	No	General health and well-being: restful sleep (IG>CG), sleep quality index during weekends (IG>CG), duration (IG>CG (ns)), daytime sleepiness (working and non-working days) (IG>CG). Mental Health: Memory Difficulty Index (IG>CG), Negative Emotions Index (IG>CG), Stress Level and Index (IG>CG), Exhaustion Index (IG>CG) and Fatigue (working and non-working days workable) (IG>CG). Work-life balance: NR
Barck-Holst ^2^ 2021 Sweden (40)	QE and qualitative	25% reduction (from 39 to 29 hours per week approx.). 18 months 2005-2006	Full-time social workers (elderly carers). N=29: IG=12; CG=17	Quantitative: one-sided t-test for independent groups comparing mean IG vs. CG. Qualitative: individual interviews.	No	General health and well-being: Qualitative: improved sleep and physical exercise among IG. Mental health: Emotional exhaustion (IG<CG), depersonalisation (IG<CG (ns)); personal accomplishment (IG>CG (ns)), less reactivity in stressful situations connected to time urgency and irritation (IG>CG). Qualitative: reduction in the feeling of exhaustion, tiredness and greater ability to recover the next day among IG (no improvements in emergencies, always very stressful). Work-life balance: Qualitative: easier to combine work with household tasks, see friends and take care of the children among IG.
Barck-Holst ^2^ 2022 Sweden (27)	Qualitative (within a QE)	25% reduction (from 39 to 29 hours per week approx.). 18 months 2005-2006	Full-time social workers with children, adolescents and families from a social work agency, low-income urban district (N=12).	Qualitative analysis with individual interviews.	No	General health and well-being: NR Mental health: Qualitative: Increased positive anticipated emotions, better recovery and lower risk of burnout. Work-life balance: Qualitative: improve balance between work and personal life, control private life demands, reduce work and life concern private, and more time for private life activities.
Costa-Font J 2021 France (28)	QE	10% reduction (from 39 to 35 hours per week) during a 46-week working year, Aubry reform (France). 4 years 1997-2006, intervention period: from 2000	White-collar and blue-collar workers of EDF-GDF (Electricité de France-Gaz de France). N=11 607; IG=11 255; CG=352	Difference in differences using linear probability models to estimate effect on IG vs. CG. Analyses adjusted for socio-demographic variables, collar and year.	No	General health and well-being: Overall self-perceived health, overweight and obesity (ns). Blue-collar workers: self-perceived health (decrease), overweight (increase), obesity (decrease). White-collar workers self-perceived health (decrease (ns), overweight (increase (ns), obesity (increase (ns). Mental health: NR Work-life balance: NR
De Spiegelaere S 2017 France (29)	Report QE	France: 10% reduction (from 39 to 35 hours per week). Time period: permanent, 1998-2008 (intervention since the beginning of the 2000s)	France: public sector, includes local administrations (NR).	France: Work-life balance. Statistical models not specified.	Yes	General health and well-being: NR Mental health: NR Work-life balance: Work-life balance positive effect reported by 58% of respondents (N=658).
De Spiegelaere S 2017 Sweden (29)	Report QE	Sweden: 25% reduction (from 8 to 6 hours per day), maintaining salary. Time period: 23 months, 2014-2016	Sweden: nursing home nurses (NR).	Sweden: IG analyses vs. CG. Statistical models not specified.	Yes	General health and well-being: self-reported good health (IG: 72%> CG: 60%), slight decrease in sickness absence (IG>CG, change ≥10%), especially nurses >50 years old. No statistical significance calculated. Mental health: Stress reduction (IG>CG, change ≥10%). No statistical significance calculated. Work-life balance: Balance work and family life ((IG>CG, change ≥10%). No statistical significance calculated.
Gomes P 2024 Portugal (30)	Report Pre-post QE	11% reduction (from 41 to 36.5 hours per week); some companies 22% (at 32 h.); 17% (at 34 hours); five started 35 hours, reduced to 34, 32, 30 or 28 hours per week. 6 months 2023	N=444; IG=332 from 41 companies (children’s home, social centre, research centre, manufacturing industry, others; questionnaire at 6 months = 2023); CG=122 from 14 companies.	IG effectiveness vs. CG, adjusted and unadjusted linear regressions for each firm’s fixed effects. Models adjusted for gender, children, educational level and salary level.	Yes	General health and well-being: Good self-perceived health (IG>CG), number of hours of sleep (IG>CG). Mental health: Good mental health (IG>CG), symptoms and anxiety (IG>CG), fatigue (IG>CG), Burnout (IG>CG). Work-life balance: Satisfaction with free time (IG>CG), reduced difficulties in balancing work and family/personal life, (IG>CG) and increased satisfaction with free time (IG>CG).
Haraldsson GD 2021 Iceland (31)	Report. Pre-post QE and qualitative	10-12% reduction (from 40 to 35/36 hours per week). Reykjavík: 5 years, 2015-2019 Government: 3 years, 2017-2020	Workers from very diverse workplaces (from offices to children’s schools, social service providers and hospitals). N=2854; IG=2500 (1% company Iceland); CG: 354 (Administration).	Quantitative: pre-post analysis among IG and CG, t-test for p-values. It does not mention analyses adjusted for other variables. Qualitative: IG and CG group interviews.	No	General health and well-being: Improvement in general health and well-being among IG. Qualitative: increased physical activity and exercise among IG. Mental health: reduction of stress symptoms among IG); single parents less stress and better time management; all statistically significant pre–post improvements among IG. Work-life balance: Work and family/personal life balance, Quality time with family, Participation in household tasks and maintenance; all statistically significant pre–post improvements among IG.
Lewis K 2023 United Kingdom (38)	Report Pre-post and qualitative evaluation (pilot)	20% reduction (from 5 to 4 days; from 40 to 32 hours per week). 6 months 2022	N=2900; IG=2900 from 61 companies marketing/advertising, services, etc. non-profit, participation: base=1,967; half=1.943; end=1.682	Quantitative: surveys and administrative data from companies, pre-post with regressions adjusted for socio-demographic and labour variables. Qualitative: group and individual interviews.	Yes (partially)	General health and well-being: decrease in sleep difficulties, improvement in wellbeing and satisfaction with life, all statistically significant pre–post improvements; no control group. Qualitative: physical and mental improvement. Mental health: improvement in mental health, burnout, fatigue, and anxiety, all statistically significant pre–post improvements; no control group. Qualitative: improvement in mental health and stress reduction. Work-life balance: improvement in reconciling work and domestic tasks, and family and social life, all statistically significant pre–post improvements; no control group. Qualitative: additional time with family and friends perceived has a significant life change.
Lindfors P 2022 Sweden (32)	QE	14% reduction (from 7 to 6 hours/day), plus health promotion activities (study circles). 12 months 2005-2006	Female carers of the elderly in the municipal service. N=87; IG=68; CG=19	Repeated-measures ANOVA analyses to assess differences over time between IG and CG (group x time interaction). Analyses adjusted for seasonal variation; groups matched at baseline.	NA (only women)	General health and well-being: self-rated health (IG>CG (ns); musculoskeletal symptoms (significant interaction, improvement in CG only); diastolic blood pressure (significant interaction, increase in CG only); HbA1c (significant interaction, decrease in CG only); DHEAS, glucose, WHR and blood lipids: significant time effects in both groups (ns for group differences). Mental health: fatigue (no differences (ns)), recovery the next day [IG<CG (NS)], perceived stress (IG>CG (ns)). Work-life balance: NR.
Mullens 2021 Belgium (33)	QE	17% reduction (from 36 to 30 hours per week). 12 months, 2018-2021 (2019 intervention)	Femma workers (non-profit women’s organisation); 1 male participant. N= 60; IG: wave 1=61; wave 2=60; wave 3=59; wave 4 =56. CG: workers 26 hours a week or less (NR).	Repeated measures ANOVA analyses, not adjusted for other variables.	NA (only women)	General health and well-being: general happiness (direction not reported (ns), sleep problems (direction not reported (NS). Mental health: reduction on mental exhaustion (improvement in all groups (ns). Work-life balance: satisfaction with the reconciliation of work and family life (IG>CG); reduced conflict between work and family life (IG>CG), greater dedication to domestic tasks and less domestic stress (IG>CG); increased leisure time, social participation and reduced leisure time pressure (IG>CG).
Mullens 2024 Belgium (34)	QE	17% reduction (from 36 to 30 hours per week. 24 months, 2018-2019 (2019 intervention)	Femma workers (non-profit women’s organisation) 1 male participant N=60; wave 1=54; wave 2=50; wave 3=51; wave 4 =45. Pre: wave 1 and 2; Post: wave 3 and 4	Multilevel growth models adjusted for other variables.	NA (only women)	General health and well-being: Warwick-Edinburgh Mental Well-being Scale [IG>CG (NS)]. Mental Health: NR. Work-life balance: decrease of work-family conflict partly explained by increased schedule control, satisfaction with work pressure and perception of sufficient free time over time.
Sanchez R 2017 France (35)	QE	10% reduction (from 36 and 39 to 35 hours) 3 years, Pre-intervention: 1994-97; Intervention: since October 1997; Post-intervention: 1998-2001	Private company workers. IG=Individuals with hours just below the old threshold and above the new one. CG (workers 30 and 35 hours per week before policy change, October 1997).	Pre-post analyses differences IG vs. CG. Probit and linear regression models adjusted for socio-economic and labour variables.	Yes	General health and well-being: self-perceived health [IG>CG (NS]). Mental health: NR. Work-life balance: NR.
Reference	Study design	Intervention & time period	Population and sample size	Analysis	Gender strat	Outcomes ^1^
Schiller ^2^ 2017 Sweden (36)	Cluster RCT	25% reduction (from 39 to 29 hours per week approx.). 18 months, 2005-2006	Full-time workers in four labour sectors (social services, technical services, care and welfare and call centre). N= 580; IG=354; CG=226	Multilevel models to evaluate differences IG vs. CG, accounting for workplace random effects (level 2) and time fixed effects (level 1), and the interaction between group and time. Models adjusted for educational level and work shifts.	Yes	General health and well-being: Improved sleep quality and duration (IG>CG), reduced sleepiness (IG>CG) and stress at bedtime (IG>CG) on weekdays. Days off: improved sleep quality (IG>CG) and sleep duration [IG<CG (NS)], reduced sleepiness (IG>CG) and stress when going to sleep (IG>CG). Mental health: Self-perceived stress (IG>CG), Worries (IG>CG) in both, working and non-working days. Work-life balance: NR
Schiller ^2^ 2018 Sweden (37)	Cluster RCT	25% reduction (from 39 to 29 hours per week approx.). 18 months, 2005-2006	Full-time workers, public sector (social services; technical services; care and welfare; call centre). N= 636; IG=370; CG=266	Multilevel models to assess differences in IG vs. CG. Models adjusted for educational level and work shifts. Subgroup analyses with Cohen’s f2, to explore variations according to gender, age, children at home and baseline levels of sleep quality and stress.	Yes	General health and well-being: NR Mental Health: NR Work-life balance: time spent on domestic tasks on working days (IG>CG), time dedicated to recovery activities (IG>CG).
Soriano E 2023 Spain (39)	Report Cross-sectional study (pilot)	20% reduction (from 40 to 32 hours per week, from 5 to 4 days per week). 3 weeks,04/10/2023 to 05/07/2023	All the workers in the city of Valencia, especially in the service sector. N=2100 participated in the post-intervention survey (not data on total participants with reduced working hours).	Survey analysis to estimate the possible impact of the reduction in working hours on health. Statistical models not specified.	Yes (partially)	General health and well-being: perceived health (IG>CG), feeling of happiness (IG>CG), tranquillity (IG>CG) and satisfaction with life (IG>CG). Mental health: reduced levels of stress and fatigue (IG>CG). Balancing work and personal life: more time for leisure activities (IG>CG), care of dependent people and personal (IG>CG).

^1^ In the quantitative studies, results are considered statistically significant unless marked as NS (not significant). ^2^ General health and well-being include variables such as general health, sleep, overweight and obesity, physical activity, and others. Mental health includes variables like stress, burnout, anxiety, and other indicators.

### Risk of bias

Quality assessment of scientific and grey literature studies is presented in supplementary table S3. The qualitative and mixed-methods studies (40, 27, 31, 38) scored 4–5, indicating high quality, and the quantitative randomized control studies (36, 37) scored 4. Quantitative non-randomized studies showed a wider variation in their quality scores, with low scores of 2 (30, 39) and 3 (29, 33, 35) and high scores of 4 (32, 34) and 5 (26, 28).

### Evidence map

The interactive evidence map is available at Evidence Map (see supplementary table S4) and displays interventions categorized into six groups based on the percentage of working hours reduction. The vertical axis represents the degree of working time reduction, while the horizontal axis groups outcomes into three categories: general health and well-being, mental health, and work–life balance. The size of the circles reflects the number of studies, and colors indicate the direction of results: green corresponds to statistically significant positive effects (or positive qualitative findings), orange to non-significant effects, and blue to mixed results within the same outcome category (ie, when a single study reported both significant and non-significant effects). No negative outcomes were identified. Available filters include benefits/support provided, continuity, public sector involvement, country, duration, sector, occupation and type of analysis (qualitative, quantitative or mixed).

### Interventions

The mapping of the interventions reported in the included literature is presented in table 2.

**Table 2:** Interventions characteristics. [HSW=health and social work.]

Ref.	Country	Percent reduction	Public sector involvement	Benefits/ support provided^1^	Duration (months)	Sector	Occupation	Continuity
26	Sweden	25	Yes	FR	18	HSW	Social workers	Temporary, continuity not planned.
40	Sweden	25	Yes	FR	18	HSW	Social workers	Temporary, continuity not planned.
27	Sweden	25	Yes	FR	18	HSW	Social workers	Temporary, continuity not planned.
28	France	10	Yes	TI	48	Industry	Blue-/white-collar	National labour reform – implemented permanently.
29	France	10	Yes	TI	120	Public administration	Administrative/ office	National labour reform – implemented permanently.
29	Sweden	25	Yes	FR	23	HSW	Nursing staff	Temporary, continuity not planned.
30	Portugal	11–17	Yes	No	6	Mix – Education, social, health	Multi-occupational	Final decisions: 90% continued: 50% adopted permanently (mainly SMEs, consulting/training, third-sector; social/educational with adaptations); 40% extended the pilot (mainly medium/large firms); 10% returned to five days (larger firms where middle management blocked organisational change).
31	Iceland	10–12	Yes	No	36	Mix – Education, social, health	Multi-occupational	Made permanent via collective agreements, 86% workforce covered (2021)
38	UK	20	No	No	6	Private services	Administrative/ office	92% continued (30% permanent); avg. reduction 38→34h/week; mainly SMEs/services, flexible models adapted by sector. 8% did not continue.
32	Sweden	14	Yes	FR	12	HSW	Nursing staff	Temporary, continuity not planned.
33	Belgium	17	No	No	12	HSW	Social workers	Temporary, continuity not planned.
34	Belgium	17	No	No	24	HSW	Social workers	Temporary, continuity not planned.
35	France	10	Yes	TI	36	Private services	Manual workers	National labour reform – implemented permanently.
36	Sweden	25	Yes	FR	18	Mix – Social, technical, welfare	Multi-occupational	Temporary, continuity not planned.
37	Sweden	25	Yes	FR	18	Mix – Social, technical, welfare	Multi-occupational	Temporary, continuity not planned.
39	Spain	20	Yes	No	<1	Public administration	Administrative/ office	Temporary, continuity not planned.

^1^ FR: Full financial reimbursement to hire additional staff; TI=Tax improvements for companies; No: there is no benefits/support offered.

*Working hours reductions.* The 15 scientific and grey literature studies described and analyzed six interventions that reduced total working hours by 10–25%. These interventions differed in both duration and implementation. Five studies from Sweden (26, 27, 36, 37, 40) analyzed a 25% reduction, resulting in a 29-hour workweek. Three studies analyzed 14–17% reductions to 30 weekly working hours (32–34). Four included studies analyzed a reduction to 35 hours: three (28, 29, 35) explored the French *Aubrey* reform (a 10% reduction to 35 hours) and one (31) examined the intervention in Iceland (a 10–12% reduction to 35/36 hours). Two studies in Spain and United Kingdom (38, 39) achieved 32-hour work weeks, representing a 20% reduction in weekly working hours. The Portuguese experiment (30) included a variety of interventions, reducing to 34, 32, 30 or 28 hours per week (11–17% reduction).

*Public sector involvement, benefits or support provided.* Of the 16 interventions reported across the 15 included studies, 13 involved government or municipal authorities, either through planning, coordination, or funding (26–31, 35–37, 39, 40). In 10 interventions, companies received some form of benefit or support: 6 studies reported full financial reimbursement to hire additional staff (26, 27, 29, 36, 37, 39) and 4 described tax incentives for participating companies (28, 29, 32, 35).

*Sectors and occupations.* Health and social work was the most represented sector, comprising five interventions focused on social workers and two on nursing staff. Four interventions included mixed sectors such as education, social services, health, technical, or welfare. Two interventions focused on public administration with administrative or office-based occupations, two were conducted in the private sector (administrative and manual workers), and one study involved the industrial sector (blue- and white-collar workers). Occupations from the primary sector (eg, agriculture and related industries) were not represented among the included studies.

*Duration.* The studies lasted between three weeks and five years (table 1), with most interventions lasting 12–24 months. In addition, all interventions were temporary, except for the French *Aubrey* labour reform, which was permanent (28, 29, 35). Three national reports (from the UK, Iceland, and Portugal) (30, 31, 38) described temporary programmes that included the possibility of becoming permanent. All interventions were mandatory for participating workers.

*Continuity.* Iceland, Portugal and the UK reports included the possibility of permanent implementation. In Iceland, reductions were made permanent through collective bargaining agreements, covering 86% of the workforce by 2021, with weekly hours reduced from 40 to 36 in the private sector, up to 32 in public services with irregular shifts (eg, hospitals, care, security), and by 65 minutes per week in public administrative/office jobs. The remaining 14% of the workforce were not covered by the collective agreements (eg, some specific private sectors or non-unionized workers) (31). In Portugal, 90% of companies continued with the reduction: 50% adopted the four-day week permanently [mainly small and medium-sized enterprises (SME)], consulting/training, and third-sector organizations, with some adaptations in social/educational settings), while 40% extended the pilot (mostly medium and large companies requiring further adjustments or additional evidence). The 10% that reverted to a five-day week were considered case-specific, primarily larger firms where middle management blocked organizational changes (30). In the UK, 92% of firms continued after the pilot (30% permanently), with an average reduction from 38 to 34 hours/week. Continuity was most common among SMEs and service-sector companies, which implemented flexible models adapted to their contexts. The 8% that discontinued cited client demands, late starts, or a preference for hybrid models (eg, 4.5 days or a 9-day fortnight) (38).

### Outcomes

The included documents reported a wide variety of outcome variables to evaluate the health impact of the interventions that have been grouped into three blocks (see table 3 and the evidence map).

**Table 3:** Summary of outcome variables by reference and classification of results. Results are classified as significant positive (⬣), not significant (⬠), significant positive results and not significant results within the same outcome (⬘). No scientific or grey literature study reported significant negative results in any outcome category. [ – =quantitative results].

Outcome category	Reference number																	Total		Positive
	(26)	(40)	(27)	(28)	(29) ^1^	(29) ^2^	(30)	(31)	(38)	(32)	(33)	(34)	(35)	(36)	(37)	(39)		N (%)		N (%)
General health and wellbeing ^3^	⬣	– ^5^	–	⬠	– ^5^	– ^5^	⬣	⬣^6^	⬣^6^	– ^5^	⬠	⬠	⬠	⬘	– ^5^	⬣		12 (75.0)		7 (58.3)
Mental health ^4^	⬣	⬘^6^	⬣^7^	– ^5^	– ^5^	⬣	⬣	⬣	⬣^6^	⬠	⬣	– ^5^	– ^5^	⬣	– ^5^	⬣		11 (68.8)		9 (81.8)
Work–life balance	– ^5^	⬣^7^	⬣^7^	– ^5^	⬣	⬣	⬣	⬣^6^	⬣^6^	– ^5^	⬣	⬣	– ^5^	– ^5^	⬣	⬣		11 (68.8)		11 (100.0)

^1^ Results from France. ^2^ Results from Sweden. ^3^ General health and wellbeing, sleep, overweight and obesity, physical activity, and other variables. ^4^ Mental health, stress, burnout and other indicators. ^5^ Quantitative results ^6^ Mixed (qualitative and quantitative) results. ^7^ Qualitative results only.

*General health and well-being.* Twelve included studies analyzed health and well-being, of which seven reported statistically significant improvements (58.3%). Mainly in self-perceived health status (29, 30, 39), well-being (29, 31), and a greater sense of happiness and life satisfaction (39). However, one study reported a slight deterioration in the perceived health of blue-collar workers (28), and the implementation of the French reform had a negative effect on the self-perceived health of young men but a positive effect on young women (35). Four studies analyzed the impact of the reduction of the working day on sleep (26, 30, 36, 38), and all of them reported improvements in the duration and/or quality of sleep, with a decrease in insomnia and sleepiness on both working days and weekends. Two studies analyzed the impact on physical activity, and both showed an increase in physical activity and exercise because of having more quality time (31, 40). Also, the French reform showed an increased overweight among blue-collar workers and decrease in obesity (28). One report included sickness absence, finding a slight but significant decrease in rates of temporary disability in nursing home nurses, especially among >50-year olds (29, 31).

*Mental health.* Among the studies reviewed, 11 included variables related to mental health (stress, fatigue, anxiety and burnout) (26, 27, 29–33, 36, 39, 40), with 9 showing significant positive results. Specifically, the stress average level and stress index both on working days and weekends (26, 29, 31, 36, 39), as well as fatigue and exhaustion index (26, 38–40), anxiety (30, 38) and recovery capacity (27, 40) showed an improvement. One of the non-positive impacts found a significant worsening in fatigue and recovery capacity (26).

*Work-life balance.* Eleven studies assessed the impact of reduced working hours on work–life balance, and all reported statistically significant improvements. Five studies found overall positive effects on work–life balance indicators (29, 31, 38, 33, 34), with one reporting a particularly stronger effect among women (30). In the Lewis study (38), 54% of participants found it easier to combine work and domestic responsibilities, leading to improvements in balancing work, family, and social life. Likewise, the proportion of workers reporting difficulties reconciling work and family demands decreased significantly (30, 33, 34). Studies focusing on predominantly female samples, such as those by Mullens et al (35, 36), observed that women who reduced their workweek from 36 to 30 hours devoted more time to housework but experienced less domestic stress and conflict between work and family life (30, 33, 34). An increase in leisure time, greater social participation, and reduced time pressure during leisure were also reported (30, 33, 34). Similarly, Schiller et al (37) found that, during workdays, intervention participants spent more time on domestic tasks but also gained an additional 53 minutes per day for recovery activities compared to controls, with no significant gender differences. In the Valencia study (39), participants—especially women—reported having more time for leisure, caregiving, and self-care. Finally, improvements were observed in life satisfaction, financial situation, personal relationships, and leisure time utilization (30).

*Qualitative data.* Qualitative data support this improvement in work–life balance (40, 27, 38) which offers workers more quality time with the family, enables better participation in (and greater ease in combining) domestic tasks, seeing friends, taking care of children, and allows for increased physical activity and exercise and improved control of private life demands. In the case of single-parent families, parents reported less stress and better time management.

### Gender analysis

Most included studies (N=11; 78.6%) included a gender perspective (see table 4), with eight reporting stratified analyses with mixed results. In the French context, three articles examined the impact of the *Aubrey* Law. Gomes et al (30) reported more favorable effects for women compared to men, particularly in mental health, work–life balance, and reductions in stress and fatigue. Sánchez (35) found improvements in self-perceived health among young women (<38 years) but a decline among young men (<39 years), potentially due to perceived reductions in career advancement opportunities immediately after the policy’s implementation. De Spiegelaere et al (29) observed that while reduced working hours promoted gender equality by decreasing part-time employment among women, women continued to bear the primary responsibility for domestic and caregiving tasks. Similar patterns were reported across other studies. Mullens et al (33, 34) analyzed a predominantly female sample (with only one male participant) and observed that, despite spending more time on housework following the reduction from 36 to 30 weekly hours, women experienced lower domestic stress and a significant decrease in work-family conflict. Soriano et al (39) reported that 51% of women and 36.6% of men increased time spent on caregiving. Specifically, men increased childcare involvement by 27% compared to a 13% increase among women. However, 68% of participants reported no substantial shift in the overall division of domestic tasks, suggesting that women continued to assume a greater share of household responsibilities. In contrast, Schiller et al (37) reported increased time devoted to domestic tasks during workdays, increased recovery time, and that individuals with children experienced reduced perceived stress, with no significant gender differences observed.

**Table 4:** Summary of the evaluation of the gender perspective in the articles. [NA=not applicable; NR=not reported].

Reference number	% of women in the sample	Outcomes stratified	Key gender findings
(26, 40, 27)	25	NA	NA
(28)	27	NA	NA
(29) ^a^	NR	Employment patterns	Not changed gender roles as women continued to bear most domestic and caregiving responsibilities. However, it helped reduce the trend of women working part-time, which positively impacted gender equality.
(29) ^b^	NR	Household division	Initiatives included a “right to work full-time,” aimed to reduce part-time, enhancing women’s employment conditions and promoting greater gender equality in the labour market.
(30)	67	Mental health, Work-life Balance, stress, fatigue	More positive impact on women, particularly in terms of mental health, work-life balance, job satisfaction, and reduced stress and fatigue.
(31)	NR	NA	NA
(38)	62	Time spent on childcare, distribution of domestic tasks	Men increased the time spent on childcare by 27%, compared to a 13% increase by women. However, the distribution of domestic tasks between men and women remained largely unchanged, with 68% of participants reporting no significant shift. These results suggest that, despite some changes in childcare involvement, women still carry a substantial share of household responsibilities.
(32)	100	NR	NA
(33, 34)	100	NR	NA
(35)	34.4	Self-perceived health	For young men (<39 years), the intervention had a negative effect on self-perceived health, particularly in the years immediately following the policy’s implementation, possibly due to perceived loss of promotion opportunities. For young women (<38 years), the intervention improved self-perceived health likely due to better work-life balance. Older men and women showed no changes in health
(36)	76	Leisure time, stress	Women did not show significantly different improvements compared to men, though they did experience a slight decrease in drowsiness on days off.
(37)	75	Domestic tasks, recovery time	Time use patterns were similar for men and women in paid work, unpaid work, and leisure activities, both on workdays and rest days, with no significant gender differences. Although women spent more time on household tasks, this did not impact the overall reduction in workload for both genders. Individuals with children reported a reduction in perceived stress.
(39)	NR	Caregiving, leisure	Stress levels were higher in young women (<45 years). Women spent more time on reading, studying, and creative activities, while men engaged more in cinema, travel, and tourism. The 36.6% of men and 51% of women dedicated more time to caregiving.

^a^ Results from France. ^b^ Results from Sweden.

### Gaps in the literature

The studies included in this review highlight the need to strengthen the scientific evidence base on working time reduction interventions. First, there is a lack of studies assessing the long-term health effects of such interventions (26, 28, 33). Second, the effects on partners or cohabitants of individuals benefiting from reduced working hours remain underexplored, particularly with regard to potential changes in household dynamics (33). Third, further research is needed to determine in which sectors and contexts these interventions can be effectively implemented (37). In addition, several papers emphasize the need for studies with larger sample sizes (26, 32–34), covering a wider range of jobs and sectors to improve generalizability (29), and involving more diverse and gender-balanced populations (27, 33).

## Discussion

This scoping review identified 16 interventions reported across 15 included studies, which were all located in high income western countries and varied in the percentage of working time reduction, duration, sector, occupation and continuity. Despite this heterogeneity, most studies reported positive effects of reducing working hours to 30–35 hours per week while maintaining salary, including improvements in general health, sleep quality, stress, burnout, and work–life balance. Some gender differences were also observed. However, the overall evidence remains limited, particularly regarding long-term effects, interventions on specific sectors of activity and occupational groups, and gendered effects. Furthermore, 5 (29, 30, 33, 34, 39) of the 15 included studies have methodological limitations and received a low-quality score; therefore their findings should be treated with caution.

The interventions included in this review reduced working hours by 10–25%, differing in duration and implementation. No clear pattern emerged between the percentage of reduction and the observed outcomes, suggesting that benefits may also depend on aspects such as other working conditions, organizational support, job redesign, and sector characteristics. Furthermore, the duration of interventions ranged from three weeks to five years, with most lasting 12–24 months. The few very long-term interventions (eg, in France) yielded mixed results, possibly influenced by contextual factors and reliance on self-reported outcomes (28, 29, 35).

Most interventions took place in the health and social work sectors, while only one industrial study was identified, and the primary sector (eg, agriculture) was not represented. Moreover, most Scandinavian interventions were implemented in the public or care sectors, where organizational structures, union coverage, and job stability differ substantially from private-sector or industrial contexts. In fact, government or municipal authorities were involved in most interventions, providing coordination, funding, or benefits to companies, such as financial reimbursement for hiring additional staff or tax incentives, highlighting the importance of public support for successful implementation (26).

Regarding impact on general health, happiness, and life satisfaction among workers with reduced working hours, results are mixed. One study found no significant changes (34), another reported a slight decline in self-reported health among blue-collar workers (28), and data from France revealed gender differences: young women showed a significant improvement in self-perceived health, whereas young men reported a decline (35). Three studies found significant improvements (29, 30, 39), however, no firm conclusions can be drawn since these studies had the lowest score in the quality evaluation. The lack of effect on self-perceived health may be related to the “healthy worker effect” (41), which could reduce the observed impact of interventions, especially in studies relying on self-reported health data.

Regarding sleep quality indicators, studies reported significant improvements (26, 40, 38, 36), including reductions in insomnia and daytime sleepiness in intervention groups. This improvement is interpreted because of reduced workload and job-related stress, as opposed to the well-documented negative impact of long working hours on sleep quality (8). Only one report included the sickness absence (30) regarding a Swedish intervention and reporting a slight but significant decrease in rates of temporary disability in nursing home nurses, especially among those aged >50 years. This aligns with several interventions promoted across European countries focused on a reduction of working hours maintaining salary for older workers (42) as from a certain age the risk of injury and sickness absence increases, especially for manual workers (43).

Of the reviewed studies, 11 included mental health variables, with most reporting significant improvements in mental health, including reduced depressive symptoms, loneliness, stress, emotional exhaustion and burnout (26, 29–33, 36, 39). Qualitative data (40, 27) support these findings, indicating a reduction in feelings of exhaustion and better daily recovery. Several hypotheses suggest that reduced working hours might lower exposure to harmful and/or stressful working conditions (40) and increase rest time (40, 27), which allows for better recovery and thus reduces exhaustion. This aligns with the concept of “external recovery” widely discussed in psychology literature (44), which posits that shorter work hours can have positive health effects by giving individuals more time to recuperate (35).

The association between working hour reductions and work–life balance indicators was reported in ten studies, with all finding significant improvements (40, 27, 29–31, 38, 33, 34, 37, 39). Notable findings include a significant increase in time dedicated to caregiving, reported more among women in two studies (33, 37) and among men in two others (38, 39). However, none of the studies showed a significant improvement in the equitable distribution of family responsibilities between men and women. In fact, it was noted that, although women reduced their working hours, this often led to an increase in domestic and childcare tasks, reinforcing traditional gender roles (33). Improved alignment of work and family responsibilities was also noted (29–31, 38, 33, 34), with one study highlighting this effect only among women (30). Increased satisfaction with time spent with family, as well as more leisure, recreational, and self-care activities, were also observed (30, 33, 34, 37, 39). This improvement may be attributed to increased time dedicated to unpaid domestic labor with the extra free time, reducing conflict between productive work and personal life, as well as schedule control.

### Strengths and limitations

This review applied a systematic and scoping methodology following PRISMA-ScR and JBI guidelines with a rigorous multi-reviewer screening and data extraction process. It comprehensively included both scientific and grey literature, allowing the identification of a broad range of interventions, some of which may not be captured by conventional academic searches. The gender perspective was explicitly incorporated throughout the review process. However, the available studies were heterogeneous in intervention designs, populations and outcomes, and several studies lacked gender-stratified analyses. Most interventions were of moderate duration (around ≥18 months) but rarely included long-term follow-up. Many studies relied on self-reported outcomes, and some studies had limited statistical power and methodological quality. Although all included studies were conducted in high-income European welfare states, contextual differences may still influence how findings can be extrapolated to other settings. Furthermore, the predominance of Scandinavian studies may also limit generalizability. These factors call for caution when interpreting the findings.

### Research priorities

Besides the gaps in the knowledge identified in the included studies, the following key aspects should be addressed in further research. Firstly, the gender perspective must be incorporated into the designs, analyses and interpretations. Stratification by sex is a necessary strategy (although not sufficient on its own) and not all included studies in this review present and explain the results stratified by sex, even the most recent reports. This omission necessarily implies a biased interpretation of the impact of working time reduction interventions on the health of the working population (45). Furthermore, this approach should be completed with an intersectional perspective (36), which considers that, beyond gender, there are other social categories and axes of inequality (such as class or race) that intersect with it to create specific exposure groups. Finally, it is essential to consider the influence of other working conditions that have a significant impact on health [such as work intensification (46) and job strain (47)] on the association between reduced working hours and the health of the working population. This is particularly critical since, as some of the included studies mentioned, there is a possibility that a reduction in the number of working hours may have an impact on work intensification, ie, working fewer but more intense hours (26, 29, 32, 33). On the other hand, it has been shown that if there is no such intensification of work, the working time reduction implies greater schedule control, which explains the improvement in work-life conflict (34).

### Concluding remarks

The reviewed studies provide several policy-relevant recommendations. Policymakers should consider supporting reduced working hours to enhance worker well-being, particularly in high-stress occupations (26). Policies should also be tailored to specific socio-economic and cultural contexts and job characteristics to ensure effectiveness across worker groups (28, 38). To maximize health benefits, long-term monitoring is recommended to allow adjustment based on observed outcomes (29). Overall, this review offers an initial perspective on the effects of working-hours reduction interventions in European contexts, identifying both emerging trends and significant knowledge gaps. Although interventions varied in design, duration, and degree of reduction, they were largely implemented in high-income European countries, limited to a narrow range of sectors and occupations, and often involved government or municipal authorities. Evidence indicates that reducing working hours to 30–35 per week without loss of pay tends to improve general and mental health and work–life balance, likely through reduced exposure to work-related stressors and increased recovery time. However, stronger evidence is needed—particularly on long-term impacts, interactions with other working conditions, sector-, occupation-, and gender-specific effects, and broader occupational coverage—supported by robust methodologies, sufficient statistical power, and extended follow-up. Record linkage studies using administrative data could help generate more conclusive evidence to guide future policy.

## Supplementary material

Supplementary material

## References

[1] Benach J, Vives A, Amable M, Vanroelen C, Tarafa G, Muntaner C. Precarious employment: understanding an emerging social determinant of health. Annu Rev Public Health 2014;35:229–53. 10.1146/annurev-publhealth-032013-182500 24641559

[2] Kivimäki M, Jokela M, Nyberg ST, Singh-Manoux A, Fransson EI, Alfredsson L et al. Long working hours and risk of coronary heart disease and stroke: A systematic review and meta-analysis of published and unpublished data for 603 838 individuals. The Lancet. 2015;386(10005):1739–46. 10.1016/S0140-6736(15)60295-1 26298822

[3] International Labour Office. Working Time and Work-Life Balance Around the World | International Labour Organization. Geneva; 2022. Available from: https://www.ilo.org/publications/working-time-and-work-life-balance-around-world

[4] International Labour Organization. Decent work and the 2030 agenda for sustainable development. [cited 2025 Oct 23]. Available from: https://www.ilo.org/topics-and-sectors/decent-work-and-2030-agenda-sustainable-development

[5] Directive - 2003/88 - EN - Working Time Directive - EUR-Lex. [cited 2025 Dec 19]. Available from: https://eur-lex.europa.eu/eli/dir/2003/88/oj

[6] Olsen KM, Dahl SÅ. Working time: implications for sickness absence and the work–family balance. Int J Soc Welf 2010 Jan;19(1):45–53. 10.1111/j.1468-2397.2008.00619.x

[7] Bobek A, Pembroke S, Wickham J. Living with uncertainty: Social implications of precarious work. Brussels; 2018.

[8] Bannai A, Tamakoshi A. The association between long working hours and health: a systematic review of epidemiological evidence. Scand J Work Environ Health 2014 Jan;40(1):5–18. 10.5271/sjweh.3388 24100465

[9] Pega F, Náfrádi B, Momen NC, Ujita Y, Streicher KN, Prüss-Üstün AM et al.; Technical Advisory Group. Global, regional, and national burdens of ischemic heart disease and stroke attributable to exposure to long working hours for 194 countries, 2000-2016: A systematic analysis from the WHO/ILO Joint Estimates of the Work-related Burden of Disease and Injury. Environ Int 2021 Sep;154:106595. 10.1016/j.envint.2021.106595 34011457 PMC8204267

[10] Garnero A. The employment effects of working time reductions in Europe. 2022 [cited 2025 Oct 13]. Available from: https://publications.jrc.ec.europa.eu/repository/handle/JRC129985

[11] European Public Service Union. Reducing working time: Case studies – 03: The Nordic countries (Sweden, Norway, Denmark, Finland). Brussels; 2024.

[12] Piasna A, Cetrulo A, Moro A. Negotiating working time reduction. 2024 [cited 2025 Oct 13]. Available from: https://www.etui.org/publications/negotiating-working-time-reduction

[13] Reports Archives - The Autonomy Institute. [cited 2025 Dec 19]. Available from: https://autonomy.work/category/reports

[14] Voglino G, Savatteri A, Gualano MR, Catozzi D, Rousset S, Boietti E et al. How the reduction of working hours could influence health outcomes: a systematic review of published studies. BMJ Open 2022 Apr;12(4):e051131. 10.1136/bmjopen-2021-051131 35365508 PMC8977802

[15] Lunau T, Bambra C, Eikemo TA, van der Wel KA, Dragano N. A balancing act? Work-life balance, health and well-being in European welfare states. Eur J Public Health 2014 Jun;24(3):422–7. 10.1093/eurpub/cku010 24567294

[16] Noda H. Work–Life Balance and Life Satisfaction in OECD Countries: A Cross-Sectional Analysis. J Happiness Stud 2020;21(4):1325–48. 10.1007/s10902-019-00131-9

[17] Moreno N, Moncada S, Llorens C, Carrasquer P. Double presence, paid work, and domestic-family work. New Solut 2010;20(4):511–26. 10.2190/NS.20.4.h 21342873

[18] Carrasquer Oto P. Tiempo de trabajo y conciliación: reflexiones para la negociación colectiva. [Working time and work-life balance: reflections for collective bargaining]Gaceta sindical: reflexión y debate. 2017;(28):251–66.

[19] Piasna A, De Spiegelaere S. Working time reduction, work–life balance and gender equality. Dynamiques régionales. 2021;10.

[20] Tricco AC, Lillie E, Zarin W, O’Brien KK, Colquhoun H, Levac D et al. PRISMA Extension for Scoping Reviews (PRISMA-ScR): checklist and Explanation. Ann Intern Med 2018 Oct;169(7):467–73. 10.7326/M18-0850 30178033

[21] Scoping Reviews - Resources | JBI. [cited 2025 Aug 19]. Available from: https://jbi.global/scoping-review-network/resources.

[22] Grey literature | Health Knowledge. [cited 2025 Aug 19]. Available from: https://www.healthknowledge.org.uk/public-health-textbook/research-methods/1a-epidemiology/grey-literature

[23] Rayyan – Intelligent Systematic Review - Rayyan. [cited 2025 Aug 19]. Available from: https://www.rayyan.ai/

[24] Hong Q, Fabregues S, Bartlett G, Boardman F, Cargo M, Dagenais P et al. The mixed methods appraisal tool (MMAT) version 2018 for information professionals and researchers. Educ Inf 2018;34(4):285–91. 10.3233/EFI-180221

[25] San Francisco. Salesforce. Tableau public. 2025 [cited 2025 May 6]. Available from: https://www.tableau.com/

[26] Barck-Holst P, Nilsonne Å, Åkerstedt T, Hellgren C. Reduced working hours and stress in the Swedish social services: A longitudinal study. 2017;60(4):897–913.

[27] Barck-Holst P, Nilsonne Å, Åkerstedt T, Hellgren C. Reduced working hours and work–life balance. Nord Soc Work Res 2022;12(4):1–14. 10.1080/2156857X.2020.1839784

[28] Costa-Font J, Saenz de Miera Juarez B. Working the Weight Out? Working Time Reduction and Overweight. Health, Econometrics and Data Group (HEDG). Working Papers. 2021;21(18).

[29] De Spiegelaere S, Piasna A. The why and how of working time reduction. Brussels: European Trade Union Institute (ETUI); 2017;88

[30] Gomes P, Fontinha R. Four-Day Week: Results from Portuguese Trial. 4 Day Week Global.; 2024.

[31] Haraldsson D, Kellam J. Going Public: Iceland’s Journey to a Shorter Working Week. Alda & Autonomy; 2021. Available from: https://autonomy.work/wp-content/uploads/2021/06/ICELAND_4DW.pdf

[32] Lindfors P, von Thiele Schwarz U. Health-related effects of an intervention involving reduced working hours among women employed in the municipal eldercare. Nord Psychol 2022;76(1):3–18. 10.1080/19012276.2022.2138516

[33] Mullens F, Verbeylen J, Glorieux I. Rethinking the workweek: results from a longitudinal time-use study of a 30-hour workweek experiment. J Time Use Res. 2021;16(1):52–75. 10.32797/jtur-2021-4

[34] Mullens F, Laurijssen I. An organizational working time reduction and its impact on three domains of mental well-being of employees: a panel study. BMC Public Health 2024 Jun;24(1):1727. 10.1186/s12889-024-19161-x 38943121 PMC11212234

[35] Sánchez R. Does a Mandatory Reduction of Standard Working Hours Improve Employees’ Health Status? Ind Relat 2017;56(1):3–39. 10.1111/irel.12163

[36] Schiller H, Lekander M, Rajaleid K, Hellgren C, Åkerstedt T, Barck-Holst P et al. The impact of reduced worktime on sleep and perceived stress - a group randomized intervention study using diary data. Scand J Work Environ Health 2017 Mar;43(2):109–16. 10.5271/sjweh.3610 27942734

[37] Schiller H, Lekander M, Rajaleid K, Hellgren C, Åkerstedt T, Barck-Holst P et al. Total workload and recovery in relation to worktime reduction: a randomised controlled intervention study with time-use data. Occup Environ Med 2018 Mar;75(3):218–26. 10.1136/oemed-2017-104592 29183947 PMC5869453

[38] Lewis K, Stronge W, Kellam J, Kikuchi L, Schor J, Fan W et al. The results are in: the UK’s four day week trial. Autonomy & 4 Day Week Global; 2023. Available from: https://autonomy.work/wp-content/uploads/2023/02/The-results-are-in-The-UKs-four-day-week-pilot.pdf.

[39] Soriano E, Ibáñez J, Hipólito F. Experiencia piloto sobre la jornada de 4 días desarrollada en València [Pilot experience on the 4-day course developed in Valencia]. Valencia; Las Naves; 2023. Available from: https://www.lasnaves.com/wp-content/uploads/2023/10/Informe_4Dies_Digital-4.pdf

[40] Barck-Holst P, Nilsonne Å, Åkerstedt T, Hellgren C. Coping with stressful situations in social work before and after reduced working hours, a mixed-methods study. Eur J Soc Work 2021;24(1):94–108. 10.1080/13691457.2019.1656171

[41] Pearce N, Checkoway H, Kriebel D. Bias in occupational epidemiology studies. Occup Environ Med 2007 Aug;64(8):562–8. 10.1136/oem.2006.026690 17053019 PMC2078501

[42] FranzEiffe, eurofoundeuropaeu, JessyeMuller, TinaWeber. Working conditions and sustainable work Keeping older workers engaged: Policies, practices and mechanisms. Dublin: European Foundation for the Improvement of Living and Working Conditions (Eurofound); 2024. Working Paper No. WPEF24030.

[43] Bláfoss R, Skovlund SV, Skals S, Sundstrup E, López-Bueno R, Calatayud J et al. Duration and intensity of occupational lifting and risk of long-term sickness absence: prospective cohort study with register follow-up among 45 000 workers. Scand J Work Environ Health 2023 May;49(4):283–92. 10.5271/sjweh.4085 36881789 PMC10713984

[44] Taris TW, Beckers DG, Verhoeven LC, Geurts SA, Kompier MA, Van Der Linden D. Recovery opportunities, work-home interference, and well-being among managers. Eur J Work Organ Psychol 2006;15(2):139–57. 10.1080/13594320500513889

[45] Valero E, Martin U, Bacigalupe A, Utzet M. The impact of precarious jobs on mental health: a gender-sensitive literature review. Int Arch Occup Environ Health 2021 May;94(4):577–89. 10.1007/s00420-020-01605-7 33236281

[46] Mauno S, Herttalampi M, Minkkinen J, Feldt T, Kubicek B. Is work intensification bad for employees? A review of outcomes for employees over the last two decades. Work Stress 2023;37(1):100–25. 10.1080/02678373.2022.2080778

[47] Niedhammer I, Bertrais S, Witt K. Psychosocial work exposures and health outcomes: a meta-review of 72 literature reviews with meta-analysis. Scand J Work Environ Health 2021 Oct;47(7):489–508. 10.5271/sjweh.3968 34042163 PMC8504166

