# Supplementary material for: Exploring the health and well-being benefits of reduced working hours with maintained salary: A scoping review and evidence map
Source: Scand J Work Environ Health. 2026 Feb 28;52(2):125–38. doi: 10.5271/sjweh.4266 (PMC12956364; doi:10.5271/sjweh.4266)

Exploring the health and well-being benefits of reduced working hours with maintained salary: A scoping review and evidence map[1]

by Mireia Utzet, PhD,[2] Mercè Soler, PhD, Jose Maria Ramada, PhD, Marta Menéndez, MSc, Michael Silva-Peñaherrera, PhD, Fernando Garcia Benavides, PhD, Consol Serra, PhD

1. Supplementary material
2. Correspondence to: Mireia Utzet, Center for Research in Occupational Health (CISAL), Universitat Pompeu Fabra, Doctor Aiguader 88, 08003 Barcelona, Spain. [E-mail: mireia.utzet@upf.edu]

Table S1. Search strategy

Database	Search strategy*
PubMed	("working time reduction" OR "reduced working hours" OR "short working hours" OR "shortened working time" OR “working time flexibility” OR "flexible work" OR "flexible working" OR "flexitime" OR "flexi time" OR "flexi-time" OR "working-time arrangements" OR “working time arrangements" OR "working-time arrangement" OR “working time arrangement" OR "part-time job" OR "part-time work" OR "work time reduction") AND (health OR well-being OR wellbeing OR "well being" OR "wellness" OR burnout OR depression OR anxiety OR pain OR QALY OR "quality of life" OR "productivity loss" OR "work-life balance" OR "sick leave" OR "time of work" OR "sickness absence" OR "presenteeism" OR "sickness leave" OR "absenteeism" OR satisfaction)
Scopus	("working time reduction" OR "reduced working hours" OR "short working hours" OR "shortened working time" OR “working time flexibility” OR "flexible work" OR "flexible working" OR "flexitime" OR "flexi time" OR "flexi-time" OR "working-time arrangements" OR “working time arrangements" OR "working-time arrangement" OR “working time arrangement" OR "part-time job" OR "part-time work" OR "work time reduction") AND (health OR well-being OR wellbeing OR "well being" OR "wellness" OR burnout OR depression OR anxiety OR pain OR QALY OR "quality of life" OR "productivity loss" OR "work-life balance" OR "sick leave" OR "time of work" OR "sickness absence" OR "presenteeism" OR "sickness leave" OR "absenteeism" OR satisfaction)
Web of Science	("working time reduction" OR "reduced working hours" OR "short working hours" OR "shortened working time" OR “working time flexibility” OR "flexible work" OR "flexible working" OR "flexitime" OR "flexi time" OR "flexi-time" OR "working-time arrangements" OR “working time arrangements" OR "working-time arrangement" OR “working time arrangement" OR "part-time job" OR "part-time work" OR "work time reduction") AND (health OR well-being OR wellbeing OR "well being" OR "wellness" OR burnout OR depression OR anxiety OR pain OR QALY OR "quality of life" OR "productivity loss" OR "work-life balance" OR "sick leave" OR "time of work" OR "sickness absence" OR "presenteeism" OR "sickness leave" OR "absenteeism" OR satisfaction)
Cochrane Library	("working time reduction" OR "reduced working hours" OR "short working hours" OR "shortened working time" OR “working time flexibility” OR "flexible work" OR "flexible working" OR "flexitime" OR "flexi time" OR "flexi-time" OR "working-time arrangements" OR “working time arrangements" OR "working-time arrangement" OR “working time arrangement" OR "part-time job" OR "part-time work" OR "work time reduction") AND (health OR well-being OR wellbeing OR "well being" OR "wellness" OR burnout OR depression OR anxiety OR pain OR QALY OR "quality of life" OR "productivity loss" OR "work-life balance" OR "sick leave" OR "time of work" OR "sickness absence" OR "presenteeism" OR "sickness leave" OR "absenteeism" OR satisfaction)
PsycINFO	("working time reduction" OR "reduced working hours" OR "short working hours" OR "shortened working time" OR “working time flexibility” OR "flexible work" OR "flexible working" OR "flexitime" OR "flexi time" OR "flexi-time" OR "working-time arrangements" OR “working time arrangements" OR "working-time arrangement" OR “working time arrangement" OR "part-time job" OR "part-time work" OR "work time reduction") AND (health OR well-being OR wellbeing OR "well being" OR "wellness" OR burnout OR depression OR anxiety OR pain OR QALY OR "quality of life" OR "productivity loss" OR "work-life balance" OR "sick leave" OR "time of work" OR "sickness absence" OR "presenteeism" OR "sickness leave" OR "absenteeism" OR satisfaction)
Epistemonikos	("working time reduction" OR "reduced working hours" OR "short working hours" OR "shortened working time" OR “working time flexibility” OR "flexible work" OR "flexible working" OR "flexitime" OR "flexi time" OR "flexi-time" OR "working-time arrangements" OR “working time arrangements" OR "working-time

	arrangement" OR “working time arrangement" OR "part-time job" OR "part-time work" OR "work time reduction") AND (health OR well-being OR wellbeing OR "well being" OR "wellness" OR burnout OR depression OR anxiety OR pain OR QALY OR "quality of life" OR "productivity loss" OR "work-life balance" OR "sick leave" OR "time of work" OR "sickness absence" OR "presenteeism" OR "sickness leave" OR "absenteeism" OR satisfaction)
ProQuest	("working time reduction" OR "reduced working hours" OR "short working hours" OR "shortened working time" OR “working time flexibility” OR "flexible work" OR "flexible working" OR "flexitime" OR "flexi time" OR "flexi-time" OR "working-time arrangements" OR “working time arrangements" OR "working-time arrangement" OR “working time arrangement" OR "part-time job" OR "part-time work" OR "work time reduction") AND (health OR well-being OR wellbeing OR "well being" OR "wellness" OR burnout OR depression OR anxiety OR pain OR QALY OR "quality of life" OR "productivity loss" OR "work-life balance" OR "sick leave" OR "time of work" OR "sickness absence" OR "presenteeism" OR "sickness leave" OR "absenteeism" OR satisfaction)
CINAHL (EBSCO)	("working time reduction" OR "reduced working hours" OR "short working hours" OR "shortened working time" OR “working time flexibility” OR "flexible work" OR "flexible working" OR "flexitime" OR "flexi time" OR "flexi-time" OR "working-time arrangements" OR “working time arrangements" OR "working-time arrangement" OR “working time arrangement" OR "part-time job" OR "part-time work" OR "work time reduction") AND (health OR well-being OR wellbeing OR "well being" OR "wellness" OR burnout OR depression OR anxiety OR pain OR QALY OR "quality of life" OR "productivity loss" OR "work-life balance" OR "sick leave" OR "time of work" OR "sickness absence" OR "presenteeism" OR "sickness leave" OR "absenteeism" OR satisfaction)

Table S2. List of government and labour agencies included for the grey literature search

Name	Region
World Health Organisation (WHO)	Worldwide
International Labour Organisation (ILO)	Worldwide
Occupational Safety and Health Administration (OSHA-EU)	European Union
Occupational Safety and Health Administration (OSHA)	United States
Social Security Administration (SSA)	United States
National Institute for Occupational Safety and Health (NIOSH)	United States
Bureau of Labour Statistics (BLS)	United States
Health and Safety Executive (HSE)	United Kingdom
Federal Agency for Occupational Risks [Agence fédérale des risques professionnels (FEDRIS)]	Belgium
Karolinska Institute. A medical University [Karolinska Institutet]	Sweden
Fair Work Ombudsman (FWO)	Australia
Heads of Workers' Compensation Authorities	Australia
Safe Work Australia	Australia
Ministry of Labour and Social Economy [Ministerio de Trabajo y Economía Social]	Spain
National Institute of Safety and Health at Work [Instituto Nacional de Seguridad y Salud en el Trabajo (INSST)]	Spain
Union of Workers' Commissions [Sindicato de Comisiones Obreras (CCOO)]	Spain
General Workers' Union [Union General de Trabajadores (UGT)]	Spain
The Work Environment Agency [Arbetsmiljöverket]	Sweden
Occupational Safety and Health Administration [Työsuojeluhallinto]	Finland
Labour Relations Authority	Ireland
Danish Agency for Labour Market and Recruitment [Styrelsen for Arbejdsmarked og Rekruttering]	Denmark
Danish Working Environment Authority [Arbejdstilsynet]	Denmark
Public Employment Service [Werkbedrijf (UWV)]	Netherlands
Netherlands Enterprise Agency [Rijksdienst voor Ondernemend Nederland]	Netherlands
Norwegian Labour and Welfare Administration (NAV)	Norway
Social Security Institute [Instituto de Segurança Social]	Portugal
Equal Employment Opportunity Commission (EEOC)	United States
Equality and human rights Commission	United Kingdom
Department of Enterprise, Trade and Employment	Ireland
Ministry of Social Affairs and Employment [Ministerie van Sociale Zaken en Werkgelegenheid]	Netherlands
Human Resources and Social Development Canada (HRSDC)	Canada
Department for Work and Pensions (DWP)	United Kingdom
Federal Institute for Occupational Safety and Health [Bundesanstalt für Arbeitsschutz und Arbeitsmedizin (BAuA)]	Germany
Ministry of Labour and Social Policies [Ministero del Lavoro e delle Politiche Sociali]	Italy
Department of Employment, Workplace Relations and Social Inclusion	Australia
Ministry of Social Affairs and Labour [Félags- og vinnumarkaðsráðuneytið]	Iceland
The Administration of Occupational Safety and Health [Vinnueftirlitið]	Iceland
Ministry of Business, Innovation and Employment	New Zealand
Ministry for Energy, Just Transition and Fair Work	Scotland
Learning and work Institute	Scotland
Ministry for Public Health and Women's Health	Scotland

Table s3. Quality assessment of scientific and grey literature studies using the Mixed Methods Appraisal Tool (MMAT)

	Screening questions		Qualitative					Quantitative randomised control					Quantitative nonrandomised					Mixed methods					Score
	S1	S2	1.1	1.2	1.3	1.4	1.5	2.1	2.2	2.3	2.4	2.5	3.1	3.2	3.3	3.4	3.5	5.1	5.2	5.3	5.4	5.5	
(26)	yes	yes											yes	yes	yes	yes	yes						5
(27)	yes	yes																yes	yes	yes	yes	yes	5
(28)	yes	yes	yes	yes	yes	yes	yes																5
(29)	yes	yes											yes	yes	yes	yes	yes						5
(30)	yes	yes											yes	yes	can't tell	can't tell	yes						3
(31)	yes	yes											no	yes	can't tell	no	yes						2
(32)	yes	yes																yes	yes	yes	can't tell	yes	4
(33)	yes	yes																yes	yes	yes	yes	yes	5
(34)	yes	yes											yes	yes	yes	no	yes						4
(35)	yes	yes											no	yes	yes	no	yes						3
(36)	yes	yes											no	yes	yes	yes	yes						4
(37)	yes	yes											can't tell	yes	can't tell	yes	yes						3
(38)	yes	yes						yes	yes	yes	can't tell	yes											4
(39)	yes	yes						yes	yes	yes	can't tell	yes											4
(40)	yes	yes											can't tell	yes	can't tell	no	yes						2

S1. Are there clear research questions? S2. Do the collected data allow to address the research questions? 1.1. Is the qualitative approach appropriate to answer the research question? 1.2. Are the qualitative data collection methods adequate to address the research question? 1.3. Are the findings adequately derived from the data? 1.4. Is the interpretation of results sufficiently substantiated by data? 1.5. Is there coherence between qualitative data sources, collection, analysis and interpretation? 2.1. Is randomisation appropriately performed? 2.2. Are the groups comparable at baseline? 2.3. Are there complete outcome data? 2.4. Are outcome assessors blinded to the intervention provided? 2.5 Did the participants adhere to the assigned intervention? 3.1. Are the participants representative of the target population? 3.2. Are measurements appropriate regarding both the outcome and intervention (or exposure)? 3.3. Are there complete outcome data? 3.4. Are the confounders accounted for in the design and analysis? 3.5. During the study period, is the intervention administered (or exposure occurred) as intended? 5.1. Is there an adequate rationale for using a mixed methods design to address the research question? 5.2. Are the different components of the study effectively integrated to answer the research question? 5.3. Are the outputs of the integration of qualitative and quantitative components adequately interpreted? 5.4. Are divergences and inconsistencies between quantitative and qualitative results adequately addressed? 5.5. Do the different components of the study adhere to the quality criteria of each tradition of the methods involved? Item 4 is not scored because it refers to the “Quantitative descriptive” study design, which did not apply to any of the included studies.

Table s4. Caption of the Evidence Map

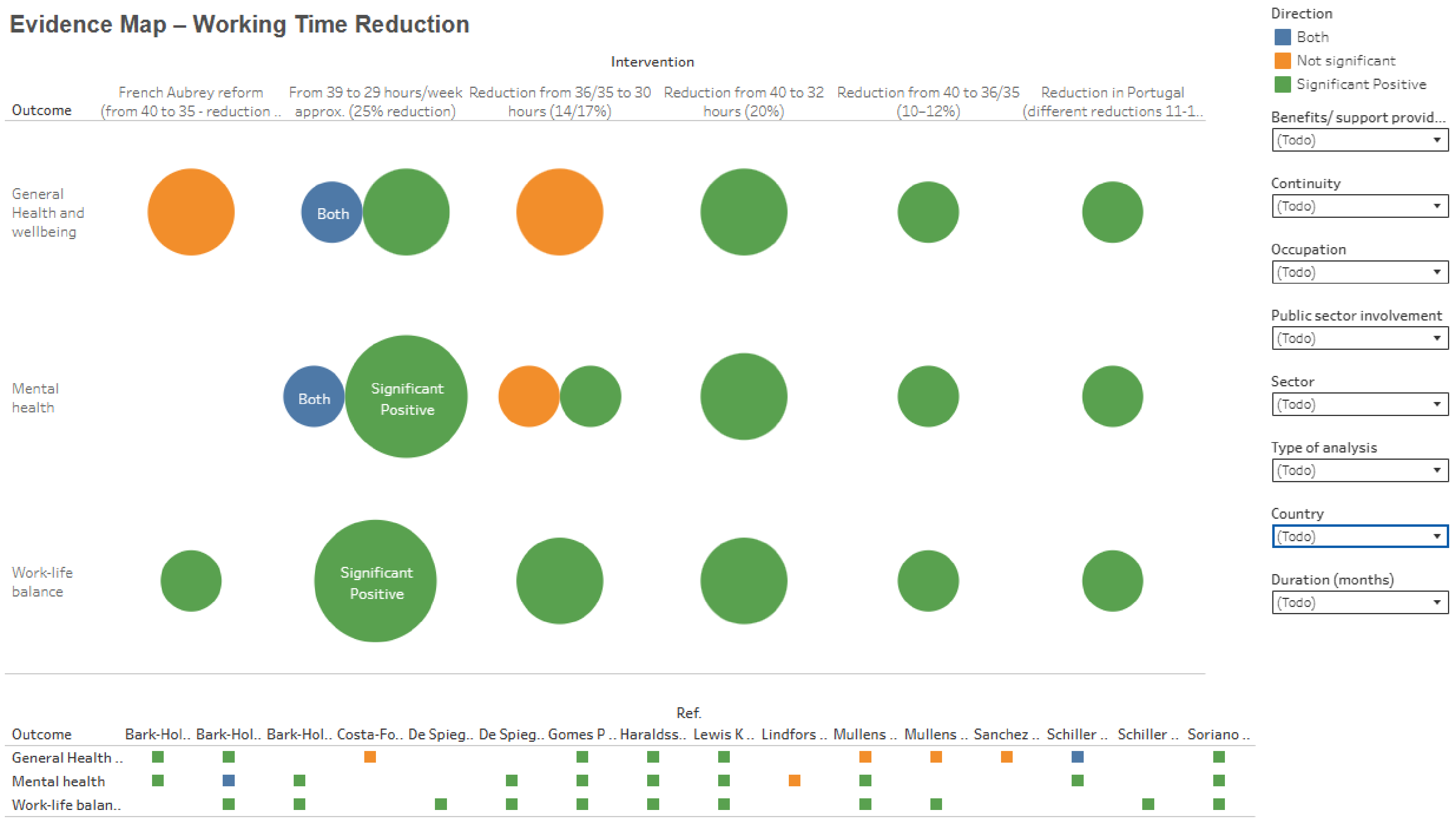

Supplement: Supplementary material [file SJWEH-52-125-S001.pdf]
